# Efficacy and safety of GLP-1 receptor agonists in Parkinson’s disease: a systematic review and meta-analysis of randomized clinical trials

**DOI:** 10.1007/s10072-026-09084-3

**Published:** 2026-05-09

**Authors:** Miguel F. Mendonça, Arthur C. Interaminense, Guilherme S. T. do R. Barros, Luciana A. Rabelo, Pedro G. de Oliveira, Rafael F. Sales, Arine M. V. de C. Lyra, Hugo R. de S. e Silva

**Affiliations:** 1 Faculdade de Ciências Médicas da Universidade de Pernambuco (FCM/UPE), R. Arnóbio Marques, 310 - Santo Amaro, Recife, PE Brasil; 2 Adjunct Professor at Faculdade de Ciências Médicas da Universidade de Pernambuco (FCM/UPE), R. Arnóbio Marques, 310 - Santo Amaro, Recife, PE Brasil; 3 Psychiatry and Medical Psychology, EPM/UNIFESP), Adjunct Professor at Faculdade de Ciências Médicas da Universidade de Pernambuco (FCM/UPE), R. Arnóbio Marques, 310 - Santo Amaro, Recife, PE Brasil

**Keywords:** Parkinson’s disease, Primary parkinsonism, Glucagon-like peptide-1 receptor agonists, GLP-1 agonists

## Abstract

**Background:**

Parkinson’s disease (PD) is a progressive neurodegenerative disorder with no proven disease-modifying therapies to date. Because changes in cerebral glucose metabolism and insulin resistance have been linked to PD pathophysiology, glucagon-like peptide-1 receptor agonists (GLP-1RAs), widely used for diabetes, have been investigated as potential neuroprotective treatments.

**Methods:**

This study systematically assessed the efficacy and safety of GLP-1RAs in PD through a systematic review and meta-analysis of randomized controlled trials identified in PubMed, Embase, and the Cochrane Library. The primary outcomes were motor function improvements measured by the MDS-UPDRS Part III in both on- and off-medication states at study endpoints and at intermediate timepoints of interest. Secondary outcomes included MDS-UPDRS Parts I, II, and IV, quality of life assessed by the PDQ-39, levodopa equivalent daily dose (LEDD), and the occurrence of adverse events.

**Results:**

The meta-analysis found no statistically significant difference in favor of GLP-1RAs over placebo for motors and non-motors outcomes, except for PDQ-39 (MD: − 0.75; 95% CI: [− 1.34, − 0.17], *P* = 0.01). Regarding safety, GLP-1RAs were associated with a higher incidence of adverse events, especially gastrointestinal effects such as nausea, vomiting, and constipation.

**Conclusions:**

Overall, current evidence does not demonstrate consistent clinical benefit of using GLP-1RAs for treating motor or non-motor symptoms in PD nor support GLP-1RAs as disease-modifying therapy, underscoring the need for further research.

## Introduction

Neurodegenerative diseases are characterized by the progressive loss of neuronal structure and function and, due to the limited regenerative capacity of nervous tissue, constitute conditions with highly complex management [1]. Although they encompass a broad spectrum, these disorders share certain elements, among which the death or inactivity of motor and/or sensory neurons and alterations in cerebral energy metabolism that contribute to neurotransmitter imbalance deserve particular emphasis [1–3]. Among them, Parkinson’s disease (PD) is one of the most prevalent worldwide, affecting approximately 13.43 per 100,000 individuals (when age-standardized), thus representing the second most common neurodegenerative disease with advancing age [4, 5].

PD is a neurological condition caused by the progressive degeneration of dopamine-producing neurons, located predominantly in the pars compacta of the substantia nigra in the midbrain [2–4, 6]. This neurotransmitter modulates motor activity within the basal ganglia; therefore, its depletion in the striatum—the basis of PD pathophysiology—leads to dysregulation of this circuit, resulting in excessive inhibition of the thalamus and, consequently, of the motor cortex, giving rise to the cardinal symptoms of the disease, namely: bradykinesia, resting tremor, rigidity, and postural instability [3, 6, 7]. Moreover, in addition to these typical motor alterations, the disease also manifests through non-motor symptoms such as hyposmia, sleep disturbances, constipation, depression, cognitive decline, among others, which often precede motor signs [3, 4, 6, 7].

In recent decades, the “gold-standard” treatment recommended for the disease has been the daily administration of levodopa (L-dopa), a dopamine precursor capable of crossing the blood–brain barrier and being converted within the central nervous system (CNS) into this neurotransmitter, thereby alleviating motor symptoms in these patients [1, 2, 7]. Nevertheless, chronic L-dopa use is associated with motor fluctuations and the emergence of dyskinesias, involuntary movements that may become disabling [2, 6]. This limitation, together with the fact that there is currently no therapy that modifies the course of the disease, constitutes the main drivers of the search for new therapeutic approaches for PD [6, 7].

In this context, recent studies involving glucagon-like peptide-1 receptor agonists (GLP-1RAs), originally used for the treatment of diabetes mellitus (DM), suggest a promising potential of these drugs in PD management [3, 6]. This hypothesis is supported, in part, by the fact that glucose is the primary energy source for nervous tissue and that alterations in its metabolism may already be present in the early stages of the disease [6, 8]. Such metabolic impairment has been related to mechanisms including insulin resistance, oxidative stress, and blood–brain barrier dysfunction, which hinder insulin signaling and promote mitochondrial dysfunction, leading to reduced efficiency of energy metabolism and negatively impacting the viability of dopaminergic neurons [8, 9]. In addition, insulin resistance is also believed to favor α-synuclein aggregation, which participates in PD pathophysiology and is notably neurotoxic [9].

Within this framework, medications in this drug class, such as lixisenatide, liraglutide, and exenatide, have recently emerged in the medical field as potentially disease-modifying agents [2, 6, 10, 11]. Among their well-described effects are stimulation of glucose-dependent insulin secretion, reduction of glucagon secretion, delayed gastric emptying, and modulation of GLP-1 receptors in the brain, reducing hunger and increasing satiety [11]. Their applicability in PD is due, in part, to this latter property, which is justified by the medication’s high penetrance across the blood–brain barrier [11]. Recent preclinical evidence in animal models indicates that, within the CNS, this agent can modulate central pathways related to insulin resistance, neuroinflammation, oxidative stress, and mitochondrial dysfunction, being effective in protecting remaining dopaminergic neurons and attenuating PD progression [2, 3, 10].

Given this promising scenario, the present study aims to systematically map and synthesize the evidence regarding the efficacy of GLP-1RAs in improving symptomatology and reducing the progression of PD, in order to discuss its therapeutic implications and identify gaps in the literature available to date.

## Methods

### Protocol and registration

This article is a systematic review, that is, a study that aims — through an explicit, critical, and systematized methodology — to synthesize primary evidence from randomized trials in order to guide health professionals’ decision-making, as well as to identify gaps in the literature, with the purpose of encouraging future investigations on a given topic [12].

The conduct and reporting of this review followed the guidelines proposed by the PRISMA 2020 (Preferred Reporting Items for Systematic reviews and Meta-Analyses) statement, a methodological strategy consisting of 27 items that guide the development of systematic reviews [13].

The protocol for this study was registered in the International Prospective Register of Systematic Reviews (PROSPERO), under the identifier CRD420251230313.

### Eligibility criteria

To compose this review, only primary studies evaluating the efficacy of GLP-1RAs in PD were included. In this context, the inclusion criteria were: (a) randomized clinical trials; (b) publication within the last 10 years (from January 2015 to September 2025); (c) intervention assessed in patients with PD diagnosed using any diagnostic tool, without demographic restrictions; (d) intervention with GLP-1 receptor agonists, with no restrictions regarding drugs within this class; and (e) studies controlled by placebo or placebo + usual care.

Therefore, the following were excluded from this review: (a) secondary studies of different types; (b) trials without results published by the date of the search, protocols, or studies still in the project phase; (c) studies that, although conducted in individuals diagnosed with Parkinson’s disease, did not evaluate the targeted intervention; and (d) studies that assessed GLP-1 receptor agonists in populations other than the one intended in this review.

### Sources of evidence and search strategy

Initially, to develop the search strategy, the research topic was delineated using the PICO strategy, establishing the population as “Individuals diagnosed with Parkinson’s disease”, the intervention as “GLP-1 agonists”, the comparison as “Placebo”, and the outcome as “Treatment efficacy and safety”.

Once the research question was defined, the search terms were established using MeSH (Medical Subject Headings), a controlled health vocabulary used to index scientific articles in databases [14]. The descriptors “Parkinson Disease” and “Glucagon-Like Peptide-1 Receptor Agonists,” as well as their synonyms, were combined with the appropriate Boolean operators to compose the final search strategy, which is detailed for each database in Table 1.

**Table 1:** Search strategy used in each database

PubMed Cochrane (MeSH)	((((((((((((((((((((Glucagon-Like Peptide-1 Receptor Agonists) OR (GLP-1 Agonists)) OR (GLP 1 Agonists)) OR (GLP1 Agonists)) OR (GLP-1 Receptor Agonists)) OR (GLP 1 Receptor Agonists)) OR (GLP1 Receptor Agonists)) OR (GLP-1R Agonists)) OR (GLP 1R Agonists)) OR (GLP1R Agonists)) OR (Incretin Mimetics)) OR (GLP-1 Analogs)) OR (GLP 1 Analogs)) OR (GLP1 Analogs)) OR (Dulaglutide)) OR (Liraglutide)) OR (Semaglutide)) OR (Exenatide)) OR (Lixisenatide)) OR (Tirzepatide)) AND (((((Parkinson Disease) OR (Parkinson's Disease)) OR (Parkinsonism)) OR (Parkinsonian Disease)) OR (Paralysis Agitans))
Embase (Emtree)	(((((((((((((((((((('glucagon-like peptide-1 receptor agonists') OR ('glp-1 agonists')) OR ('glp 1 agonists')) OR ('glp1 agonists')) OR ('glp-1 receptor agonists')) OR ('glp 1 receptor agonists')) OR ('glp1 receptor agonists')) OR ('glp-1r agonists')) OR ('glp 1r agonists')) OR ('glp1r agonists')) OR ('incretin mimetics')) OR ('glp-1 analogs')) OR ('glp 1 analogs')) OR ('glp1 analogs')) OR ('dulaglutide')) OR ('liraglutide')) OR ('semaglutide')) OR ('exenatide')) OR ('lixisenatide')) OR ('tirzepatide')) AND ((((('parkinson disease') OR ('parkinson`s disease')) OR ('parkinsonism')) OR ('parkinsonian disease')) OR ('paralysis agitans'))

Given that the Embase search system differs from the others, the query translator tool available within the database itself was used to adapt the search strategy to the platform’s search interface

### Study selection

The retrieved articles were exported to the Rayyan software, an online tool that allows organizing, selecting, identifying, grouping, and excluding articles exported from databases in an individual and precise manner [15].

After duplicate removal, an initial screening of the articles was performed by three independent reviewers, who blindly assessed the titles and abstracts of these studies to identify those potentially meeting the previously established eligibility criteria. Subsequently, full-text reading of these records was conducted for inclusion in the present study. Disagreements were resolved based on the decision of a fourth reviewer, who did not participate in either screening phase. The process was carried out independently among reviewers to minimize selection bias, allowing for the impartial inclusion of studies based solely on the eligibility criteria described above.

### Outcomes of interest

The primary outcomes of this study included the assessment of motor function using the Movement Disorder Society–Unified Parkinson’s Disease Rating Scale, Part III (MDS-UPDRS Part III) [16], measured both in the on-medication and off-medication states. These measures were analyzed at two different timepoints: a 6-month midpoint and an endpoint which corresponds to the actual final follow-up time in the included trials, allowing comparison of motor performance evolution throughout participant follow-up. The timepoints that were pooled together are presented in Table 2.

**Table 2:** Timepoints of data collection throughout the included studies

ID	Intervention	Tp 1	Tp 2	Tp 3	Tp 4	Tp 5	Tp 6	Tp 7
Athauda et al. [17]	Exenatide	12w	24w #	36 w	48 w	60 w *	-	-
Hogg et al. [18]	Liraglutide	4w	12w	26w	28w #	38w	52w	54 w *
McGarry et al. [19]	NLY01	2w	3w	4w	12w	24w #	36w *	-
Meissner et al. [20]	Lixisenatide	2w	24w #	48w *	-	-	-	-
Vijiaratnam et al. [21]	Exenatide	24w #	48w	72w	96w *	-	-	

*Tp* timepoint, *W* weeks, # Considered midpoints of interest and pooled together; * Considered endpoints and pooled together

Secondary outcomes included MDS-UPDRS Parts I, II, IV, the Parkinson’s Disease Questionnaire (PDQ-39), the levodopa equivalent daily dose (LEDD), and the incidence of adverse events.

### Data extraction

A standardized data extraction table was developed by the authors in accordance with the objective of the present study. All reviewers participated in the extraction phase. Data from each study were extracted independently by two reviewers and, for each included article, another reviewer was responsible for verifying the extracted data, ensuring that the information was consistent with the respective studies. In studies where discrepancies in collected data occurred, these were assessed and resolved by that reviewer.

Regarding study characteristics, the following were extracted: identification (last name of the first author and year of publication), trial registration number, study design, country, duration and follow-up checkpoints, sample size, intervention and comparator (as well as dose and route of administration), outcomes assessed, and the main findings of each study.

Concerning participant characteristics, the following were extracted: intervention, sample, age (in years), sex (as male/female), time since diagnosis, and the baseline score on MDR-UPDRS Part I, II, III and IV, the PDQ-39 scale, and the levodopa equivalent daily dose (LEDD).

For continuous outcomes in which the mean or standard deviation were not reported by the studies, the authors estimated these values from available data (e.g., medians, interquartile ranges, upper and lower limits, etc.) using validated conversion methods [22–24].

### Risk-of-bias assessment

The quality of the studies included in this review was assessed independently by two reviewers using the Cochrane Risk-of-Bias Tool 2 (RoB 2). RoB 2 comprises five domains: (1) bias arising from the randomization process; (2) bias due to deviations from intended interventions; (3) bias due to missing outcome data; (4) bias in outcome measurement; and (5) bias in selection of the reported result. For each domain, studies were categorized according to the level of risk, being classified as low risk, some concerns, or high risk of bias [25].

### Statistical analysis and effect measurement

The meta-analysis was conducted using Review Manager (RevMan) 5.4 [26]. To estimate the pooled effects of GLP-1 receptor agonists vs. placebo, continuous data were extracted as mean, standard deviation (SD), and number of participants (n) in each group, enabling calculation of the mean difference (MD) with a 95% confidence interval (95% CI). The authors considered there was no need to use the standardized mean difference (SMD) to estimate effects because the studies used the same scales to assess the outcomes. Conversely, dichotomous outcomes were expressed as risk ratios (RR), also with 95% CI. For meta-analysis of continuous variables, the inverse-variance model was used, whereas for dichotomous variables, the Mantel–Haenszel method was applied.

Because the intervention (GLP-1 receptor agonists) comprises a group of medications that includes different drugs, the authors anticipated that there could be high heterogeneity among studies; therefore, random-effects models were used to estimate the pooled effect. However, for the outcome “adverse effects,” a fixed-effects model was used due to the similarity of these events among the different medications within this class of medicines.

Heterogeneity was assessed qualitatively through analysis of the Forest Plot generated by the software and quantitatively using I^2^. An I^2^ value ≥ 40% was considered an indicator of high heterogeneity, prompting a sensitivity analysis, which was performed by excluding articles from the pooling one at a time. Findings with *p* < 0.05 were considered statistically significant.

Furthermore, for trials including multiple intervention arms with different doses, each dose was treated as an independent comparison against the shared placebo group. To avoid unit-of-analysis errors, the placebo group was proportionally divided when necessary, in accordance with Cochrane recommendations.

## Results

### Study selection

**Fig. 1:**
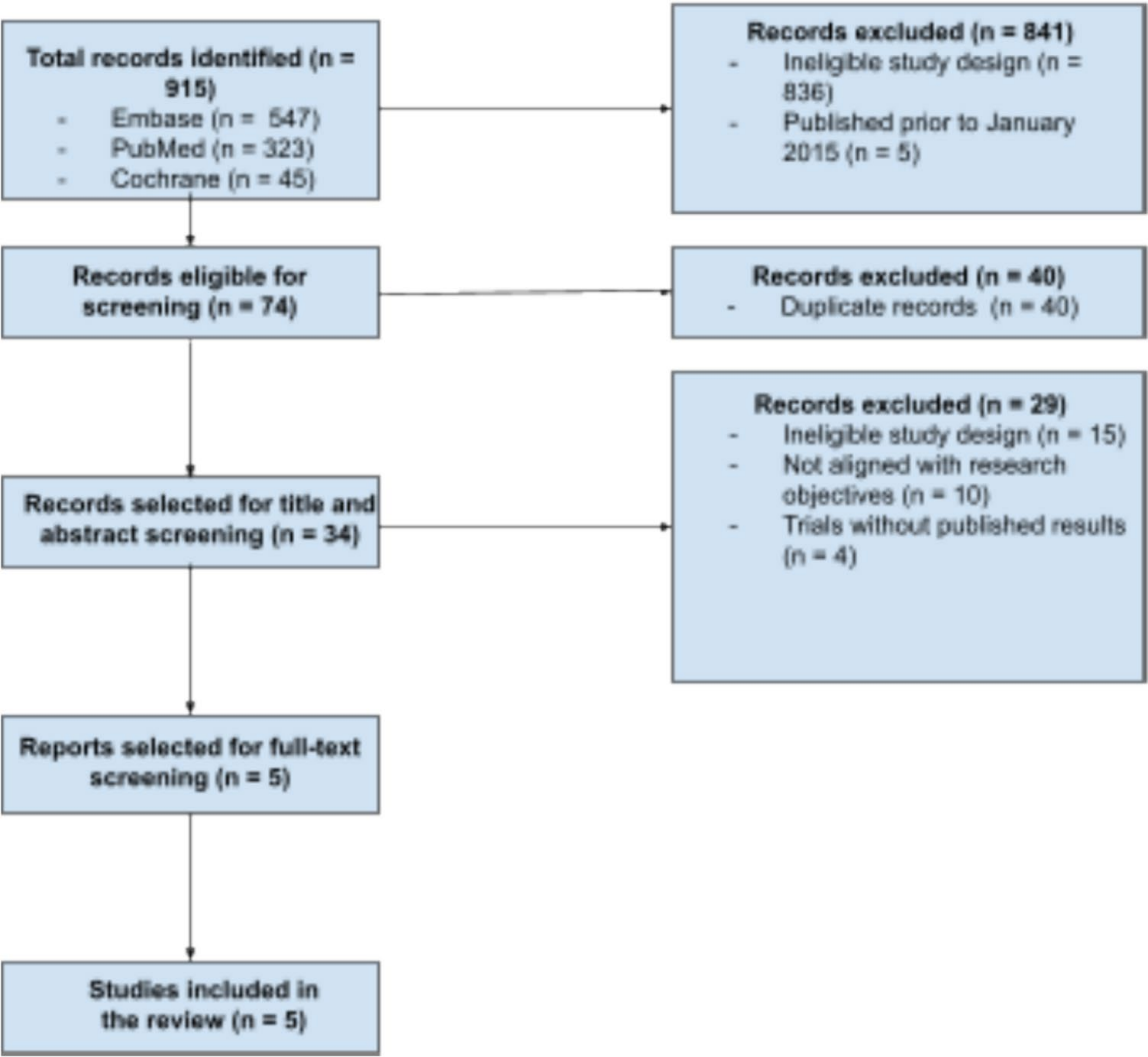
Flowchart indicating the article selection process

In our research 915 records were retrieved from the databases and, after the initial application of the eligibility criteria, 74 randomized clinical trials (RCTs) proceeded to screening. After removal of 40 duplicates, 34 studies underwent title and abstract screening to identify studies potentially eligible for inclusion. Of these, 5 were subjected to full-text review and fully met the eligibility criteria of the present study, and were therefore included in the analysis. The step-by-step study selection process is detailed in Fig. 1.

### Characteristics of the included studies and participating individuals

Five RCTs met the previously established eligibility criteria and were therefore included in this review [17–21]. All included trials were double-blind and, collectively, randomized 730 participants, with individual sample sizes ranging from 62 [17] to 255 [19] subjects. The mean age of participants allocated to the studies ranged from 57.8 to 64.2 years, and in four of the five studies [17–20] most randomized individuals were male. All individuals included in the five studies had a prior diagnosis of PD and, with the exception of participants in McGarry et al. [19], all were using L-dopa as usual treatment. Only one study [19] presented an intervention group with two arms using different doses and, likewise, only this study had a duration shorter than 12 months. For meta-analytic purposes, each dose arm was treated as an independent comparison against the placebo group.

The GLP-1RAs assessed across the included studies comprised exenatide [17, 21], liraglutide [18], NLY01 (a long-acting pegylated analog of exenatide) [19], and lixisenatide [20]. In all included studies, medications were administered via subcutaneous injections, with doses and the total administration period varying across studies. The characteristics of the included studies are presented in Table 3, and baseline participant characteristics are grouped in Table 4.

**Table 3:** Summary of the included studies

ID	Trial register	Study design	Country	Follow-up duration	Sample size	Intervention
Athauda et al. [17]	NCT01971242	Double-blind RCT	United Kingdom	0w; 12w; 24w; 36w; 48w; 60w	62	Exenatide
Hogg et al. [18]	NCT02953665	Double-blind RCT	United States of America	0w; 4w; 12w; 26w; 28w; 38w; 52w; 54w	63	Liraglutide
McGarry et al. [19]	NCT04154072	Double-blind RCT	United States of America	0w; 2w; 3w; 4w; 12w; 24w; 36w	255	NLY01
Meissner et al. [20]	NCT03439943	Double-blind RCT	France	0w; 2w; 24w; 48w	156	Lixisenatide
Vijiaratnam et al. [21]	NCT04232969	Double-blind RCT	United Kingdom	0w; 24w; 48w; 72w; 96w	194	Exenatide

Comparator	Dose and route	Outcomes assessed	Main findings/conclusions
Placebo + UC	2 mg SC weekly for 48w	MDS-UPDRS Part III MDS-UPDRS Part I MDS-UPDRS Part II MDS-UPDRS Part IV NMSS MADRS Mattis-DRS PQD-39 EQ-5D LEDD Adverse events	Although it is not yet clear whether exenatide affects the underlying pathophysiology of the disease or simply produces long-lasting effects, the treatment showed positive effects on defined motor scores in patients with Parkinson’s disease, and these effects persisted beyond the exposure period
Placebo + UC	1.2 mg SC once + 1.8 mg SC weekly for 51w	MDS-UPDRS Part III MDS-UPDRS Part I MDS-UPDRS Part II MDS-UPDRS Part IV NMSS MDRS-2 PDQ-39 DKEFS Escala de Depressão Geriátrica PAS HbA1C HOMA-IR Adverse events	Therapy with liraglutide showed a significant improvement in non-motor symptoms, activities of daily living (ADLs), and broader quality-of-life measures compared with placebo, particularly in NMSS scores. Adverse effects were mostly gastrointestinal, along with weight loss and transient elevations in liver and pancreatic enzyme levels
Placebo	2.5 mg SC weekly or 5 mg SC weekly for 36w	MDS-UPDRS Part II MDS-UPDRS Part III MDS-UPDRS Part I MDS-UPDRS Part IV CGI-S PGI-S SE-ADL PDQ-39 MoCA SCOPA-Cog NMSS DaTScan Adverse events	NLY01 was not effective, as it did not differ from placebo at either dose (2.5 mg and 5 mg). Adverse events were mainly gastrointestinal and were more frequent with the higher dose
Placebo + UC	10 mcg SC daily for 2w + 20 mcg SC daily for 46w	MDS-UPDRS Part III MDS-UPDRS Part I MDS-UPDRS Part II MDS-UPDRS Part IV PQD-39 MoCA LED Adverse events	Treatment with lixisenatide resulted in less progression of motor disability than placebo, but it was associated with gastrointestinal side effects
Placebo + UC	2 mg SC weekly for 96w	MDS-UPDRS Part III MDS-UPDRS Part I MDS-UPDRS Part II MDS-UPDRS Part IV MoCA UDysRS NMSS PDQ-39 EQ-5D PHQ-9 LEDD	The main finding was that exenatide treatment did not show a significant benefit in improving motor function or slowing symptom progression in early-stage Parkinson’s disease in this phase 3 trial, unlike the phase 2 study. Gastrointestinal side effects were also reported

*RCT* randomized clinical trial, *W* weeks, *UC* usual care, *mg* milligrams, *mcg* micrograms, *SC* subcutaneously, *MDS-UPDRS* movement disorder society–unified Parkinson’s disease rating scale, *NMSS* non-motor symptoms scale, *MADRS* montgomery-asberg depression rating scale, *Mattis-DRS* Mattis dementia rating scale, *PQD-39* 39 item Parkinson's disease questionnaire, *EQ-5D* EuroQol- 5 dimension, *LEDD*, levodopa equivalent dose, *MoCA* montreal cognitive assessment, *UDysRS* unified dyskinesia rating scale, *PHQ-9* patient health questionnaire-9, *MDRS-2* Mattis dementia rating scale, second edition, *DKEFS* Delis-Kaplan executive function system. *PAS* Parkinson anxiety scale, *CGI-S* clinical global impression – severity, *PGI-S* patient global impression of severity, *SE-ADL* Schwab and England activities of daily living, *SCOPA-Cog* scales for outcomes in Parkinson's disease cognition, *DaTScan* dopamine transporter scan

**Table 4:** Baseline characteristics of included studies’ participants

ID	Group	N	Age	Sex	Time since diagnosis	MDS-UPDRS I	MDS-UPDRS II	MDS-UPDRS III	MDS-UPDRS IV	LEDD
Athauda et al. [17]	Exenatide	31	61.6 (8.2)	22/9	6.4 (3.3)	9.8 (4.8)	12.5 (6.7)	32.8 (9.7) - OFF 19.4 (8.4) - ON	4.7 (3.1)	773.9 (260.9)
	Placebo	29	57.8 (8.0)	22/7	6.4 (3.3)	9.2 (3.8)	10.7 (5.3)	27.1 (10.3) - OFF 14.4 (8.2) - ON	5.3 (3.0)	825.7 (215.0)
Hogg et al. [18]	Liraglutide	42	63.5 (9.8)	25/12	-	7.9 (4.8)	8.8 (5.4)	26.1 (9.6) - OFF 14.8 (7.1) - ON	3.8 (3.3)	564 (327)
	Placebo	21	64.2 (6.4)	13/5	-	6.4 (4.1)	7.6 (5.0)	28.8 (10.7) - OFF 16.3 (9.2) - ON	3.6 (3.2)	640 (360)
McGarry et al. [19]	NLY01 2.5 mg	85	62.1 (9.0)	60/25	1.01 (1.03)	4.2 (3.1)	4.8 (3.6)	22.7 (8.1) - OFF (-) ON	-	-
	NLY01 5 mg	85	60.6 (10.0)	54/31	0.96 (0.93)	4.0 (3.7)	5.0 (4.1)	22.0 (8.2) - OFF (-) ON	-	-
	Placebo	84	61.8 (8.1)	52/32	0.89 (0.99)	4.7 (4.2)	4.9 (3.6)	22.3 (9.1) - OFF (-) ON	-	-
Meissner et al. [20]	Lixisenatide	78	59.5 (8.1)	44/34	1.4 (0.8)	6.1 (4.0)	5.0 (3.5)	(-) OFF 14.8 (7.3) - ON	0.3 (1.3)	317 (179)
	Placebo	78	59.9 (8.4)	48/30	1.4 (0.7)	6.4 (4.2)	5.4 (4.3)	(-) OFF 15.5 (7.8) – ON	0.2 (0.8)	355 (215)
Vijiaratnam et al. [21]	Exenatide	97	61.02 (9.05)	69/28	-	7.9 (4.9)	7.4 (4.9)	32.2 (12.5) - OFF 20.0 (10.2) - ON	3.9 (3.3)	
	Placebo	97	60.35 (9.26)	69/28	-	7.5 (4.8)	7.5 (4.9)	32.3 (13.3) - OFF 20.8 (10.4) - ON	3.8 (3.2)	

*N* sample size, *MDS-UPDRS* movement disorder society–unified Parkinson’s disease rating scale, *LEDD* levodopa equivalent daily dose. All values in parentheses correspond to the SD of the means, which values are presented outside the parentheses. Age, as well as time since diagnosis, are reported as mean years. Sex is expressed as male/female proportion. MDS-UPDRS scores and LEDD are presented as the mean values of these measures within the population included in each group

### Risk-of-bias of the included studies

**Fig. 2:**
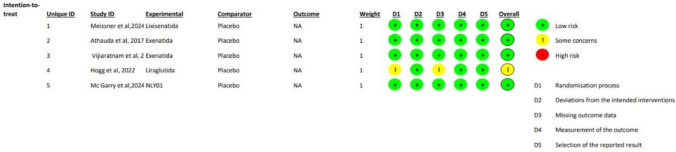
Risk-of-Bias assessment

The risk-of-bias assessment, conducted using the Cochrane RoB 2, indicated that there was no risk of bias in any of the studies regarding deviations from intended interventions, outcome measurement, and selection of the reported results. Four of the five included studies were considered to be at low risk of bias [17, 19–21], whereas one study [18] raised some concerns in the overall judgment due to limitations in the randomization process and missing reported data. No studies were classified as being at high risk of bias. A summary of the risk-of-bias assessment is presented in Fig. 2.

### Primary outcomes

#### MDS-UPDRS part III “off-medication” at endpoint

**Fig. 3:**
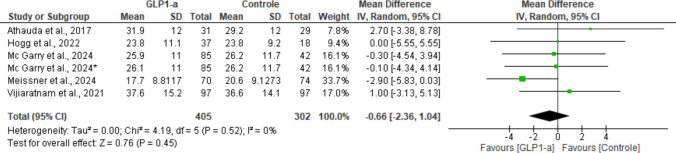
Analysis of the MDS-UPDRS Part III off-medication outcome at the end of the studies

This outcome was assessed by all included studies [17–21]. Our analysis concluded that there was a statistically non-significant difference between groups (MD: − 0.66; 95% CI: [− 2.36, 1.04], *P* = 0.45) regarding changes in motor symptoms at the end of the trials, with no evident heterogeneity across studies (I^2^ = 0%; *P* = 0.52) (Fig. 3).

One of the trials [19] did not report the standard deviations of the final scores for this outcome. Thus, for analytical purposes, the final values were estimated from the available baseline data and the mean changes over follow-up. The standard deviations of the changes were derived from the standard errors and sample size and were then combined with the baseline SD, assuming independence between measures, as recommended by Cochrane [22]. This method is conservative and tends to overestimate variance, thereby reducing the risk of optimistic bias in the effect estimates [22].

In addition, for all outcomes, the McGarry et al. trial [19] was divided into three groups, namely placebo, 2.5 mg intervention, and 5 mg intervention. In the figures, when this article appears without an asterisk, the results refer to the group that received the lower dose, whereas the asterisk indicates the group in which the higher dose was administered. Across individual analyses, neither dose arm showed statistically significant differences compared with placebo.

#### MDS-UPDRS part III “off-medication” at midpoint

**Fig. 4:**
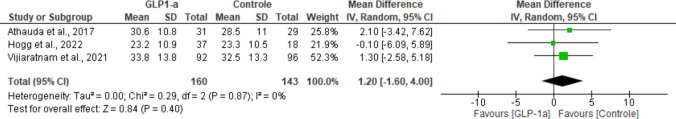
Analysis of the MDS-UPDRS Part III off-medication outcome at a midpoint of the studies

The pooled analysis of the three studies that reported data for this outcome [17, 18, 21] did not reveal significant differences between groups (MD: 1.20; 95% CI: [− 1.60, 4.00]; *P* = 0.40). Also, no heterogeneity was identified across studies (I^2^ = 0%; *P* = 0.87) (Fig. 4).

#### MDS-UPDRS part III “on-medication” at endpoint

**Fig. 5:**
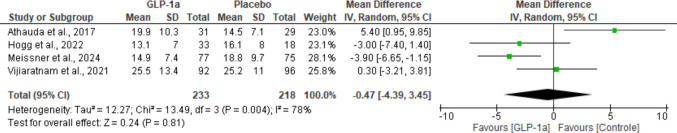
Analysis of the MDS-UPDRS Part III on-medication outcome at the end of the studies

Four of the five included studies [17, 18, 20, 21] reported outcome data on MDS-UPDRS Part III in the “on-medication” state. Meta-analysis of these data showed a statistically non-significant improvement among patients treated with GLP-1RAs (MD: − 0.47; 95% CI: [− 4.39, 3.45], *P* = 0.81) (Fig. 5).

Due to the high heterogeneity identified (I^2^ = 78%; *P* = 0.004), a sensitivity analysis was performed, which showed that removing the study by Athauda et al. [17] led to a slight reduction in heterogeneity across studies (I^2^ = 43%; *P* = 0.18). However, even after removal of this study, the difference between groups remained statistically non-significant (MD: − 2.30; 95% CI: [− 4.95, 0.36], *P* = 0.09).

#### MDS-UPDRS part III “on-medication” at midpoint

Data on this outcome were reported by four of the included studies [17, 18, 20, 21]. The estimated effect showed no significant difference between patients in the intervention group and those in the control group (MD: 0.42; 95% CI: [− 2.36, 3.20]; *P* = 0.77). The heterogeneity observed across studies was high (I^2^ = 50%; *P* = 0.11).

**Fig. 6:**
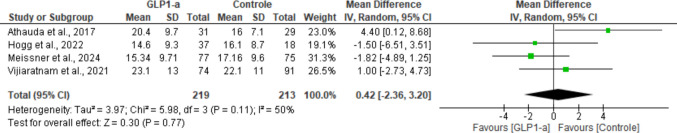
Analysis of the MDS-UPDRS Part III on-medication outcome at a midpoint of the studies

In one of the studies [20], the absolute values of MDS-UPDRS Part III in the “on” state at the intermediate timepoint of interest were not reported directly. Therefore, for analytical purposes, they were estimated from the baseline mean and the change from baseline, as recommended by Cochrane [22] (Fig. 6).

Sensitivity analysis showed that exclusion of the article by Athauda et al. [17] markedly reduced heterogeneity (I^2^ = 0%; *P* = 0.50); however, the outcome remained statistically non-significant (MD: − 0.83; 95% CI: [− 2.97, 1.31]; *P* = 0.45).

### Secondary outcomes

#### MDS-UPDRS part I

**Fig. 7:**
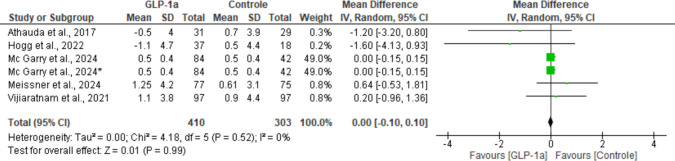
Analysis of the MDS-UPDRS Part I outcome at the end of the studies

All included studies reported data related to the change from baseline for this outcome. The pooled analysis revealed no statistically significant difference between groups (MD: 0.00; 95% CI: [− 0.10, 0.10]; *P* = 0.99). Heterogeneity across studies was null (I^2^ = 0%; *P* = 0.52) (Fig. 7).

#### MDS-UPDRS part II

**Fig. 8:**
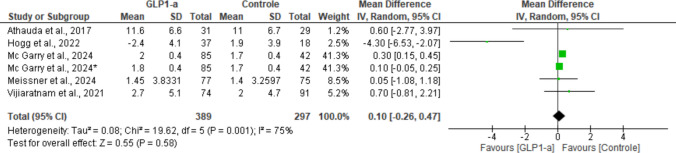
Analysis of the MDS-UPDRS Part II outcome at the end of the studies

Changes from baseline in MDS-UPDRS Part II scores were reported by all studies. There was no significant difference between the group that received the intervention and the group that received placebo (MD: 0.10; 95% CI: [− 0.26, 0.47]; *P* = 0.58). For this outcome, the studies showed high heterogeneity (I^2^ = 75%; *P* = 0.001) (Fig. 8).

A sensitivity analysis was performed and showed that removal of the study by Hogg et al. [18] markedly reduced heterogeneity (I^2^ = 1%; *P* = 0.40). Moreover, after removal of this study, the outcome became statistically significant (MD: 0.20; 95% CI: [0.10, 0.31]; *P* = 0.0002) and favored the control group.

#### MDS-UPDRS part IV

**Fig. 9:**
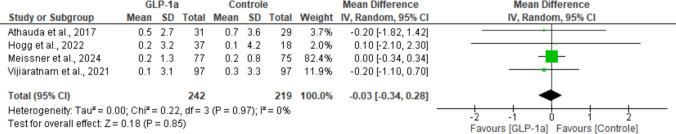
Analysis of the MDS-UPDRS Part IV outcome at the end of the studies

Only one of the included studies did not report data for this outcome [19]. The estimated effect was statistically insignificant for the difference between groups (MD: − 0.03; 95% CI: [− 0.34, 0.28]; *P* = 0.85). There was no heterogeneity across studies (I^2^ = 0%; *P* = 0.97) (Fig. 9).

#### PDQ-39

**Fig. 10:**
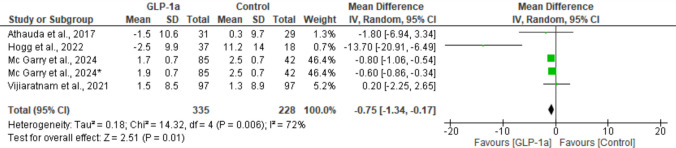
Analysis of the PDQ-39 outcome at the end of the studies

Of the five studies, four reported data regarding the PDQ-39 [17–19, 21]. Although the study by Meissner et al. [20] described the change in the PDQ-39 score as an exploratory outcome, the corresponding values were not presented in the main article, and thus it was not possible to include this study in the quantitative synthesis. Data analysis revealed a small but statistically significant difference favoring the GLP-1RA group (MD: − 0.75; 95% CI: [− 1.34, − 0.17], *P* = 0.01). For this outcome, the studies showed high heterogeneity (I^2^ = 72%; *P* = 0.006) (Fig. 10).

A sensitivity analysis was performed in the meta-analysis, and it was observed that removal of the article by Hogg et al. [18] markedly reduced heterogeneity (I^2^ = 0%; *P* = 0.61). Thus, after removal of this study, the outcome remained statistically significant in favor of the GLP-1RA group (MD: − 0.70; 95% CI: [− 0.88, − 0.51]; *P* < 0.00001).

#### LEDD

**Fig. 11:**
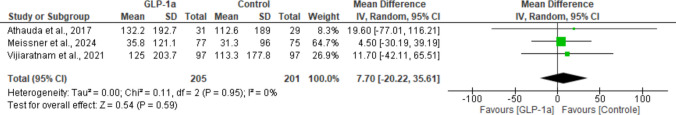
Analysis of the LEDD outcome at the end of the studies

Of the four studies that set out to assess this outcome [17, 18, 20, 21], one [18] could not be included in the quantitative analysis due to the absence of raw data presented in the body of the trial. Thus, this study was excluded from the quantitative synthesis. The pooled analysis revealed statistically non-significant differences for LEDD (MD: 7.70; 95% CI: [− 20.22, 35.61]; *P* = 0.59) and no heterogeneity across studies (I^2^ = 0%; *P* = 0.95) (Fig. 11).

In the study by Vijiaratnam et al. [21], the change in LEDD over the 96 weeks of the trial was reported as median and interquartile range. To allow inclusion of these data in the meta-analysis, these values were converted into mean and standard deviation. Means were estimated using the method described by Luo et al. [24], whereas standard deviations were estimated according to the method of Wan et al. [23], both providing a conservative estimate of variance to avoid overestimating values, as recommended by Cochrane [22].

In addition, in the study by Athauda et al. [17], exogenous dopaminergic exposure was described as levodopa equivalent dose (LED), according to a previously recommended standardization. However, according to Tomlinson et al. [27], the LED reported by the study could be considered operationally equivalent to the levodopa equivalent daily dose (LEDD), since the study meets the six methodological criteria established for this equivalence, and therefore the values were included in the quantitative analysis.

## Adverse events

### Nausea

**Fig. 12:**
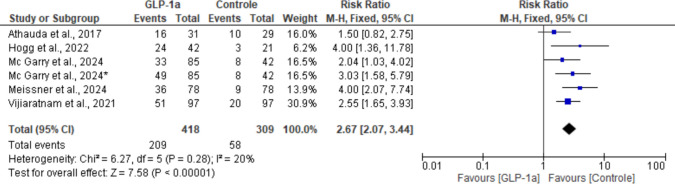
Analysis of the Nausea outcome at the end of the studies

All five studies reported data for this outcome. The pooled analysis suggests a strong correlation between GLP-1RAs and this adverse effect, which is corroborated by the high statistical significance of the mean difference for this outcome (RR: 2.67; 95% CI: [2.07, 3.44]; *P* < 0.00001). A small heterogeneity was identified across studies (I^2^ = 20%; *P* = 0.28) (Fig. 12).

### Vomiting

**Fig. 13:**
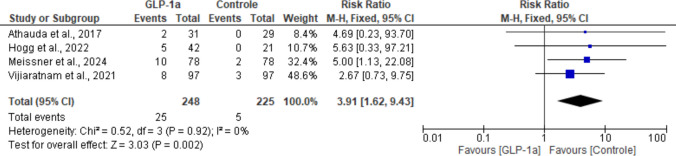
Analysis of the Vomiting outcome at the end of the studies

All five trials reported data for this outcome, which was more frequent in the experimental population. However, one study [19] was not included in the pooled analysis due to a single event in the control group, which could not be appropriately distributed after splitting the control arm to avoid double counting. According to our analysis, a statistically significant difference favoring the control group was observed (RR: 3.91; 95% CI: [1.62, 9.43]; *P* = 0.002), with no heterogeneity across studies (I^2^ = 0%; *P* = 0.92) (Fig. 13).

### Constipation

**Fig. 14:**
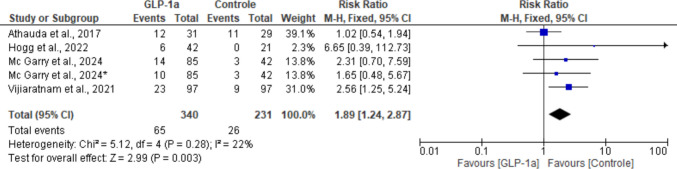
Analysis of the Constipation outcome at the end of the studies

All studies reported this adverse effect. Consistent with the previous safety outcomes, the difference between groups was statistically significant, favoring the control group and thus suggesting a correlation between GLP-1RAs and constipation (RR: 1.89; 95% CI: [1.24, 2.87]; *P* = 0.003). A small heterogeneity was observed across studies (I^2^ = 22%; *P* = 0.28) (Fig. 14).

### Diarrhea

**Fig. 15:**
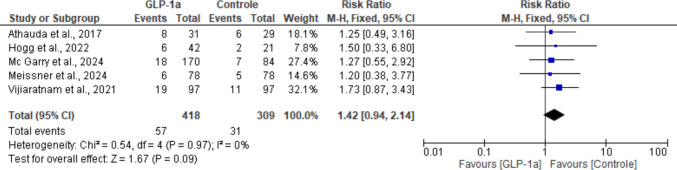
Analysis of the Diarrhea outcome at the end of the studies

Diarrhea was also more frequent in the experimental group; however, the between-group difference favoring placebo was statistically insignificant (RR: 1.42; 95% CI: [0.94, 2.14]; *P* = 0.09). There was no heterogeneity across studies for this outcome (I^2^ = 0%; *P* = 0.97) (Fig. 15).

In this analysis, the two intervention arms from the McGarry et al. study [19] were combined into a single group. This approach was adopted because the control group presented an odd number of events, which could not be evenly distributed when splitting the control arm to avoid double counting. Therefore, combining the intervention arms was considered the most appropriate strategy to preserve data integrity.

### Anxiety

**Fig. 16:**
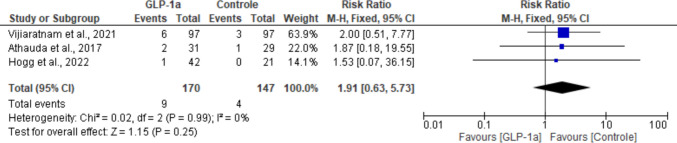
Analysis of the Anxiety outcome at the end of the studies

All five studies reported anxiety as being more frequent in the experimental group compared with the control group. Our analysis showed statistical non-significance favoring placebo (RR: 1.91; 95% CI: [0.63, 5.73]; *P* = 0.25). There was no heterogeneity across studies for this outcome (I^2^ = 0%; *P* = 0.99) (Fig. 16).

### Weight loss

**Fig. 17:**
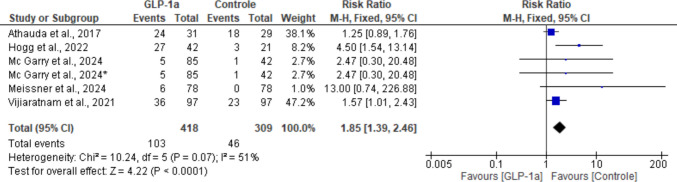
Analysis of the Weight Loss outcome at the end of the studies

All five studies reported weight loss as an adverse event, being more frequent in the experimental group compared with the control group. Our analysis showed statistical significance for the between-group difference for this outcome (RR: 1.85; 95% CI: [1.39, 2.46]; *P* < 0.0001). The studies also showed a high heterogeneity (I^2^ = 51%; *P* = 0.07), possibly due to differential effects among the medications with respect to weight loss (Fig. 17).

A sensitivity analysis was performed and showed that removal of the article by Hogg et al. [18] markedly reduced heterogeneity (I^2^ = 12%; *P* = 0.34), however, the outcome remained statistically significant in favor of the control group (RR: 1.61; 95% CI: [1.20, 2.16]; *P* = 0.001).

## Discussion

### Findings

The correlation between type 2 diabetes mellitus and Parkinson’s disease (PD) has been investigated for a long time, given the worse motor and non-motor outcomes experienced by patients with diabetes compared with those without diabetes, as well as the more rapid deterioration of cognitive function also observed in these individuals [28]. This connection between degenerative diseases that, at first glance, share little similarity in symptomatology motivated the development of studies in orthotopic PD models aimed at testing the influence of GLP-1RAs on the progression of this α-synucleinopathy [29]. In these studies, this drug class demonstrated a neuroprotective potential in animal models, reflected by preservation of dopaminergic neurons and attenuation of neuroinflammatory processes such as oxidative stress and mitochondrial dysfunction, translating into improvements in pathological markers [29].

These findings, together with the lack of therapeutic strategies capable of modifying the natural course of the disease and the progressive loss of efficacy of established symptomatic medications for PD as the disease advances, motivated the conduct of clinical trials designed to determine the efficacy of GLP-1RAs across different outcomes observed in individuals with PD [10]. This systematic review with meta-analysis examined primary data from these studies regarding the efficacy of this class of antidiabetic drugs on motor and non-motor symptoms of PD, and also investigated the safety profile of these medications based on the incidence of adverse events.

In light of our findings, despite a sensible biological and preclinical rationale, the aggregated results do not robustly support a clinically relevant improvement in motor function in PD patients treated with GLP-1RAs. This is evidenced by the absence of a statistically significant mean difference between the experimental and control groups on the MDS-UPDRS Part III scale both in the on-medication and off-medication states, whether at intermediate assessment timepoints or at study endpoints. Importantly, the trial by McGarry et al. [19], which evaluated two different doses of NLY01, did not demonstrate differential effects between the 2.5 mg and 5 mg arms. This lack of gradient suggests the absence of a clear dose–response relationship within the studied range, reinforcing the overall finding of limited efficacy of GLP-1RAs in PD.

Moreover, the lack of statistical significance for improvement in MDS-UPDRS Part II scores (reflecting activities of daily living) in the intervention group raises another important consideration: even if the medications produce a modest benefit, it does not translate into functional gains in everyday life, a factor of critical importance in PD. In this context, a disease-modifying intervention, beyond affecting motor examination scores, should confer greater autonomy to the patient, minimize the risk of falls, improve gait, reduce caregiver burden, and so forth — effects that were not observed with GLP-1RAs.

Regarding quality of life, although a significant improvement in PDQ-39 scores was observed, this did not parallel the magnitude of improvement in motor outcomes, as assessed by the UPDRS Part III, nor in functional outcomes measured by Part II. This dissociation suggests that the observed benefit in patient-reported outcomes is not directly driven by motor or functional changes. While a potential effect on non-motor symptoms may be hypothesized, this remains speculative given the lack of domain-specific PDQ-39 data and the absence of other dedicated non-motor assessments across studies.

Alternatively, a placebo contribution cannot be fully excluded, particularly considering the subjective nature of quality-of-life measures, as these outcomes may be influenced by patients’ expectations of improvement, which have been associated with activation of reward-related dopaminergic pathways in the ventral striatum. Moreover, the small magnitude of the observed effect raises questions regarding its clinical relevance. These findings should therefore be interpreted with caution, especially given the heterogeneity across studies and limitations in available data.

A crucial point in interpreting the discrepancy between the clear improvements in animal models and the results observed in human clinical trials is understanding the heterogeneity of PD. Although the core of the disease is midbrain dopaminergic degeneration, PD is an α-synucleinopathy with an inflammatory component of the nervous parenchyma, encompassing mitochondrial dysfunction, oxidative stress, impaired glucose metabolism, insulin resistance, and additionally influenced by other patient comorbidities. Because the samples in the included studies were highly heterogeneous, with participants at different disease stages and with varying severities of motor and non-motor symptoms, even if a benefit of GLP-1RAs exists, it may not have been detected.

Another important factor that may contribute to the observed heterogeneity is the pharmacokinetic and pharmacodynamic variability among the GLP-1 receptor agonists evaluated. Although these agents share a common mechanism of action, they differ substantially in terms of half-life, blood–brain barrier penetration, receptor affinity, and dosing frequency. For instance, exenatide and its long-acting analog NLY01 are structurally distinct from liraglutide and lixisenatide, which may influence their central nervous system exposure and neuroprotective potential. Moreover, differences in molecular size and albumin binding may affect drug distribution and duration of action, potentially impacting clinical outcomes. Therefore, pooling these agents as a homogeneous class may obscure drug-specific effects and partially explain the lack of consistent efficacy observed in this meta-analysis.

Furthermore, the statistical non-significance of the difference in levodopa equivalent daily dose (LEDD) suggests that GLP-1RAs were ineffective at reducing the need for clinically meaningful adjustments in dopaminergic drugs, which would be expected if the intervention had a robust disease-modifying effect or a sustained symptomatic effect.

Finally, from a safety perspective, the pooled analyses indicate that adverse events associated with GLP-1RAs are not marginal. A consistent increase in adverse effects, especially gastrointestinal events, was observed, raising concerns regarding the use of this drug class in PD. This is because PD frequently already involves constipation, slowed intestinal transit, nausea, and related symptoms, and a drug that exacerbates these effects may worsen treatment adherence, nutritional status, and even the response to L-dopa, whose absorption occurs in the gastrointestinal tract. In addition, weight loss was frequently observed. While weight reduction represents a targeted therapeutic outcome in the treatment of diabetes and obesity — primary indications for GLP-1RAs [30] — it warrants caution in the context of PD. In these patients, weight loss may aggravate the physical frailty inherent to disease progression, with the potential to negatively influence long-term functional capacity and quality of life.

### Agreements and disagreements with other studies

From a methodological standpoint, this review differs from prior papers by representing the most up-to-date synthesis on the topic, incorporating five published randomized clinical trials, the most recent of which was published in February 2025. In addition, some previously published reviews adopted less specific approaches by aggregating a broader range of antidiabetic medications, which may increase clinical heterogeneity and hinder inferences specifically directed at GLP-1RAs, the objective of the present study.

Regarding the primary outcome assessing motor function in patients with PD, our results align with evidence from recent reviews, such as Abou Ellez et al. [31] and Nogueira et al. [32], in indicating the absence of a robust motor improvement in PD and the need for additional trials to define the true clinical value of GLP-1RAs.

In contrast, our effect estimate differs from the findings of Albuquerque et al. [33], Messak et al. [34], and Zhang et al. [35], which reported significant motor benefits — a discrepancy that may be attributable to variations in the search time window, inclusion criteria, or handling of timepoints.

It is noteworthy that, although studies such as Mulvaney et al. [36] and Wang et al. [37] described favorable effects, these conclusions derived from a quantitative analysis of evidence with low certainty and high risk of bias, which contrasts with the consistency of the clinically non-significant results observed in our analysis, whose overall risk of bias was considered low. In addition, one study concluded that exenatide specifically can promote cognitive benefits [37], an outcome that was not evaluated in our review.

A statistically significant improvement in PDQ-39 favoring the intervention group suggests that, even without a robust motor effect, there may be clinical impact in patient-centered domains. This finding complements the existing literature and reinforces the importance of future studies exploring, in greater depth, functional and quality-of-life outcomes, as well as potential subgroups with a higher likelihood of response.

Finally, regarding safety, our results are consistent with prior literature in indicating an increased risk of, particularly, gastrointestinal adverse events associated with GLP-1RAs [31, 34], reinforcing that tolerability remains a critical component for interpreting effectiveness and for designing new trials.

### Limitations

Several limitations should be considered when interpreting the findings of this study. Although the available body of evidence presents a low risk of bias, it is relatively limited, with RCTs of small sample sizes, which reduces the statistical power of the analysis. In addition, considerable heterogeneity was observed across multiple outcomes, possibly related to differences in participant characteristics (such as age, time since diagnosis, and disease severity), therapeutic regimens, the specific GLP-1RA studied, and concomitant use of different antiparkinsonian medications, which limits direct comparability across studies and the robustness of the estimated effects.

Moreover, the duration of follow-up across included studies ranged from 36 to 96 weeks, which may be insufficient to adequately assess long-term neuroprotective effects. Given the typically slow progression of Parkinson’s disease, longer follow-up is necessary to detect meaningful changes in disease trajectory. Additionally, variability in follow-up durations across studies may have contributed to heterogeneity in the observed outcomes. Therefore, future trials with extended follow-up are warranted to better clarify the potential neuroprotective role of GLP-1 receptor agonists.

Another important point is that, even when statistical significance was observed in some scenario, the clinical relevance of the finding may be questionable, since very small effects may fail to reach a minimally important threshold for patients, suggesting that any associated benefits may be subclinical or dependent on specific assessment conditions. It should also be considered that treatment adherence may have influenced results, since in all trials the medication was self-administered subcutaneously, introducing uncertainty because this route is less common in the general population and may cause pain in more sensitive patients. Additionally, most studies did not track key biomarkers, such as the exosomal protein ⍺-synuclein within extracellular vesicles, whose aggregation is central to the pathogenesis of PD, and there’s evidence suggesting that monitoring its levels may be useful in managing the disease’s progression [38, 39].

In parallel, the small number of studies limits subgroup analyses (by disease stage, symptom severity, age, etc.), restricting generalizations about who would benefit most and under which conditions the response would be more consistent. Finally, it was not possible to fully rule out publication bias, especially because, with few studies, formal tests (such as Egger’s test) become unreliable, leaving open the possibility of underreporting of unpublished trials due to negative results.

## Conclusions

This systematic review with meta-analysis of five RCTs concluded that there is no consistent evidence of benefit from GLP-1 receptor agonists that would justify their recommendation as symptomatic and/or disease-modifying therapy for patients with Parkinson's disease in routine clinical practice. In addition, use of this drug class was robustly associated with a higher frequency of adverse events, particularly gastrointestinal events, compared with placebo. Conversely, factors such as the short duration of follow-up across some studies, the use of different drugs among the trials, as well as the heterogeneity of the analyzed outcomes may have influenced the results and limit definitive conclusions. Therefore, to better understand the true efficacy of GLP-1RAs in PD, further studies with larger sample sizes, more homogeneous populations, longer follow-up, and incorporating the assessment of PD biomarkers are needed.

## Data Availability

All data generated or analysed during the current study are included in this published article.
